# Possible Effect of Polycystic Ovary Syndrome (PCOS) on Cardiovascular Disease (CVD): An Update

**DOI:** 10.3390/jcm13030698

**Published:** 2024-01-25

**Authors:** Nicia I. Profili, Roberto Castelli, Antonio Gidaro, Roberto Manetti, Margherita Maioli, Marco Petrillo, Giampiero Capobianco, Alessandro P. Delitala

**Affiliations:** 1 Department of Medicine, Surgery, and Pharmacy, University of Sassari, 07100 Sassari, Italy; nicia.isa.profili@gmail.com (N.I.P.); rcastelli@uniss.it (R.C.); rmanetti@uniss.it (R.M.); mpetrillo@uniss.it (M.P.); capobia@uniss.it (G.C.); 2 Department of Biomedical and Clinical Sciences Luigi Sacco, University of Milan, Luigi Sacco Hospital, 20122 Milan, Italy; gidaro.antonio@asst-fbf-sacco.it; 3 Department of Biochemical Science, University of Sassari, 07100 Sassari, Italy; mmaioli@uniss.it

**Keywords:** polycystic ovary syndrome, PCOS, hyperandrogenism, cardiovascular disease, CVD, hypertension, arterial stiffness, atherosclerosis

## Abstract

Polycystic ovary syndrome (PCOS) is the most common endocrine disorder in women during the fertile period. Women with PCOS have an increased risk of developing major cardiovascular risk factors during the fertile period: obesity, impaired glucose tolerance, diabetes mellitus, dyslipidemia, and metabolic syndrome. The possible effect of PCOS on cardiovascular disease (CVD) has been reported in different studies, but the results are not clear for several reasons. Indeed, most of the studies analyzed a cohort of fertile women who, given their relatively young age, have a low frequency of cardiovascular diseases. In addition, longitudinal studies have a short follow-up period, insufficient to draw firm conclusions on this topic. Finally, pharmacological treatment is limited by the lack of specific drugs available to specifically treat PCOS. In this review, we report on studies that analyzed the possible effect of PCOS on the most common CVD (hypertension, arterial stiffness, atherosclerosis, and cardiovascular event) and available drugs used to reduce CVD in PCOS women.

## 1. Introduction

**Figure 1:**
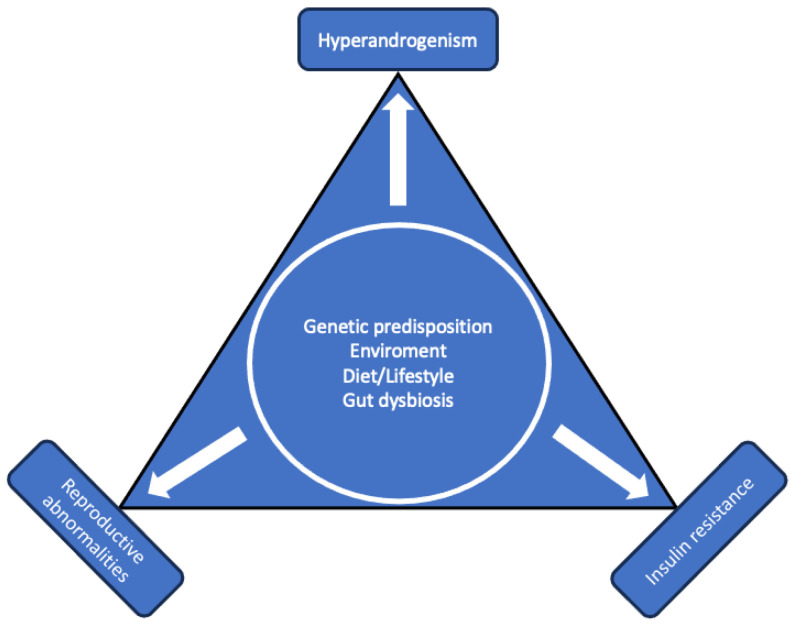
Pathophysiology of PCOS.

Polycystic ovary syndrome (PCOS) is the most common endocrine disorder in women during the fertile period [1]. PCOS is a heterogenous disease whose prevalence varies according to the different studies with a clear genetic and geographical effect [2]. PCOS has a complex and not completely understood pathophysiology (Figure 1), and the diagnosis is based on specific criteria due to the lack of information on its causes.

The first attempt to standardize PCOS diagnosis was performed by a small group of experts during the 1990 National Institutes of Child Health and Human Development (NICHD) Conference on PCOS and later became known as the National Institute of Health (NIH) criteria. These included clinical or biochemical hyperandrogenism and chronic anovulation (after the exclusion of secondary causes) [3]. Later, in 2003, a Consensus Conference in Rotterdam highlighted the importance of polycystic ovarian morphology, which was added as a diagnostic criterion of PCOS [4]. The presence of three criteria allowed for the addition of three additional phenotypes (polycystic ovarian morphology + hyperandrogenism, polycystic ovarian morphology + ovulatory disfunction, and polycystic ovarian morphology + hyperandrogenism + ovulatory disfunction) to the single PCOS phenotype with the NIH criteria (ovulatory disfunction + hyperandrogenism). In 2006, the Androgen Excess Society suggested to consider the presence of hyperandrogenism as a required component for the diagnosis of PCOS, thus reducing the clinical phenotypes [5]. In 2012, the PCOS consensus workshop group reviewed and summarized the current knowledge of PCOS [6]. Recently, in 2023, Teede et al. published the new recommendations from the international evidenced-based guidelines for the diagnosis of PCOS [7]. This study suggested a diagnostic algorithm in which PCOS diagnosis was possible in the presence of clinical hyperandrogenism and irregular menses, the latter after excluding other causes of oligo-amenorrhea. Women without clinical hyperandrogenism should be further tested for biochemical hyperandrogenism after excluding secondary causes of increased androgen production (e.g., Cushing’s syndrome and adrenal tumors). In the case of the presence of only irregular menses or hyperandrogenism, the algorithm suggested a specific approach based on the woman’s age: adults required an ultrasound to assess the polycystic ovarian morphology, while adolescents did not need ultrasound and may be reassessed later.

## 2. Method of Search Strategy

A detailed literature search on MEDLINE, Cochrane library, Pubmed, and Google Scholar databases was performed up to December 2023 with restriction to the English language. The keywords used for this review were the following: “Polycystic ovary syndrome”, “PCOS”, “cardiovascular disease”, “hypertension”, “arterial stiffness”, “atherosclerosis”, “intima-media thickness”, “IMT”, “cardiovascular event”, and “treatment of PCOS”. Original articles, reviews, and meta-analyses were included. The most significant and relevant articles were included in this study.

### 2.1. PCOS and Hypertension

Hypertension is the most common cardiovascular disease in the general population, whose prevalence progressively increases during aging [8]. The European Society of Cardiology defined hypertension as the presence of office systolic blood pressure values ≥ 140 mmHg and/or diastolic blood pressure values ≥ 90 mmHg [9].

Several studies compared blood pressure (absolute values as well as prevalence of hypertension) in PCOS women and healthy controls with different results (Table 1). Meun et al. analyzed 200 middle-aged women and showed that hypertension was more prevalent in women with PCOS compared to age-matched controls [10]. Similarly, the Northern Finland Birth Cohort 1966 Study showed that PCOS women had higher blood pressure (systolic and diastolic) compared to controls, even at an early age and independently of BMI [11]. Another study reported that, while the values of both systolic and diastolic blood pressure are increased in PCOS women compared to controls, the prevalence of hypertension was comparable between groups [12].

Other authors analyzed the relation between PCOS and 24 h ambulatory blood pressure measurement (ABPM). The study was conducted in 60 Turkish women (mean age 30.5 years) with a previous diagnosis of PCOS according to the Rotterdam Criteria and without a history of hypertension. The authors reported that daytime blood pressure (both systolic and diastolic) and nighttime diastolic blood pressure were significantly higher in PCOS women compared to healthy controls. The study further highlighted a higher frequency of masked hypertension in PCOS women (36.6% vs. 24.4%, *p* = 0.009) [13]. Joham et al. reported the result of a secondary analysis of the Australian Longitudinal Study on Women’s Health [14] by conducting a Cox proportional hazards model to identify predictors of blood hypertension. The study, which analyzed over 9500 women for 15 years, found that 16.4% of the women developed hypertension, whose incidence was significantly higher in PCOS woman compared to women without. The analyses showed that women with PCOS had a 37% greater risk of hypertension, which increased in cases of obesity.

Albeit most of the studies reported the analysis of PCOS women in the fertile period, few studies analyzed the association between hypertension and PCOS after menopause. Schmidt et al. reported the results of a prospective follow-up study on 35 postmenopausal women who were re-evaluated 21 years after the baseline visit [15]. The authors reported a higher frequency of hypertension, which did not increase the risk of cardiovascular events during the follow-up. A recent systematic review and meta-analysis showed that the OR for hypertension was increased in menopausal women with a previous diagnosis of PCOS compared to controls (OR 1.79). Conversely, another meta-analysis by Amiri et al. reported that the risk of hypertension in PCOS women was increased only in reproductive age and not after menopause [16]. A key role might be played by hyperandrogenemia, in particular after menopause. This hypothesis has been tested on an experimental rat model in which chronic exposure to higher levels of androgens led to the development of insulin resistance, visceral obesity, and hypertension [17].

The relation between hypertension and PCOS has also been studied in pregnant women. Bahri Khomani et al. analyzed data from the Australian Longitudinal Study on Women’s Health, including 5838 pregnant women [18]. Univariate analysis showed that PCOS was associated with a higher incidence of hypertensive disorders. However, in the multivariate analyses adjusted for confounders, PCOS was no longer associated with hypertension; the subgroup analysis clearly showed that the risk was significantly higher only in non-obese women. Another study, somewhat in line with the previous results, reported that the presence of PCOS in pregnant women did not affect systolic and diastolic blood pressure values or the prevalence of hypertension [19]. However, a study by Lønnebotn et al. reported that PCOS was related to hypertensive disorders in pregnancy (RR 1.62) in women with a specific body weight [20]. Indeed, while the risk was reported to be increased in underweight and obese women, it was found to be insignificantly increased in normal weight and overweight women.

Several mechanisms have been identified to explain the association between PCOS and hypertension. For example, Cascella et al. reported increased levels of aldosterone in PCOS women compared to healthy controls (10.5 vs. 5.7 ng/dl) [21]. As stated by the same authors, aldosterone levels were within the normal range in both groups. However, even aldosterone within the physiological range may lead to hypertension [22]. In addition, aldosterone significantly correlated with fasting insulin concentration in PCOS women. These data were somewhat confirmed in a previous study, which found an increased prevalence of metabolic syndrome in primary hyperaldosteronism [23]. Another possible mechanism that might explain the increased frequency of hypertension in PCOS women is an imbalance in the autonomic nervous system caused by the compensatory hyperinsulinemia found in cases of insulin resistance, which could also cause increased renal sodium reabsorption and decreased nitric oxide production [24].

Overall, the studies are not consistent in the association between hypertension and PCOS. Although studies reported a slight increase in blood pressure values in PCOS women, its real clinical significance still needs to be clarified.

**Table 1:** Case-control studies on hypertension in women with polycystic ovary syndrome (PCOS).

Study	Type of Study	n		Mean Age (yrs)	Mean Follow-Up (yrs)	Results
		PCOS	Controls			
De Jong [25]	Cross-sectional	24	29	38.4	N/A	No difference in blood pressure
Johan [14]	Prospective	681	7542	21–42	15	Increased incidence rate of hypertension independently of BMI
Khomami [18]	Prospective	492	5346	30.5	3	Higher risk of hypertension in non-obese women
Kałużna [26]	Cross-sectional	249	85	24.9	N/A	Higher blood pressure level (but within the normal ranges) in PCOS women with hyperandrogenism
Mellembakken [12]	Cross-sectional	512	281	28.0	N/A	Higher blood pressure within the normal values in PCOS
Wu [27]	Retrospective	20,652	82,608	29.0	N/A	Increased risk of developing hypertension in PCOS, which is increased in presence of diabetes mellitus and dyslipidemia
Özkan [13]	Cross-sectional	60	60	26.4	N/A	Higher masked hypertension in PCOS
Hey Hg [28]	Longitudinal	199	242	30.6	10.6	Increased frequency of hypertension in PCOS
Meun [10]	Cross-sectional	200	200	50.5	N/A	Higher prevalence of hypertension in PCOS
Behboudi-Gandevani [29]	Prospective	178	1524	26.4	12.9	Higher risk of developing hypertension in PCOS but the risk was diluted in late reproductive period
Ollila [11]	Cross-sectional	577 ^#^	62 ^#^	31	N/A	Higher blood pressure values in PCOS and higher frequency of hypertension independently of overweight/obesity
		765 ^#^	100 ^#^	46	N/A	
		336 ^##^	95 ^##^	31	N/A	
		698 ^##^	137 ^##^	46	N/A	
Glintborg [30]	Prospective	1165	52,769	29	11.1	Increased rate of hypertension in PCOS
Bairey Merz [31]	Prospective	25	270	62.6	5.9–9.3	No difference in blood pressure value and frequency of hypertension
Chang [32]	Cross-sectional	117	204	41	N/A	Increased risk of hypertension, independently of race
Joham [33]	Cross-sectional	478	8134	N/A	N/A	Increased prevalence of hypertension in PCOS, not associated to BMI
Shi [34]	Cross-sectional	3396	1891	30.49	N/A	Increased frequency of hypertension in PCOS

Abbreviations: PCOS, polycystic ovary syndrome; N/A, not applicable; yrs, years; ^#^ Normal weight; ^##^ Overweight/obese.

### 2.2. PCOS and Arterial Stiffness

Arterial stiffness can be assessed with different methods, each of which has specific characteristics and limits [35]. Pulse wave velocity (PWV) is a measurement of arterial stiffness and represents the velocity at which the pulse of blood pressure propagates through the vessels. It is calculated as the distance of a specific arterial segment divided by the time taken for the pulse to travel on that segment. Among the methods created to assess PWV, the most widely used is the carotid–femoral PWV (cf-PWV), which consists of a collection of signals from the carotid and femoral arteries simultaneously performed or sequentially guided with ECG-gated. Although cf-PWV is the most reliable method used to evaluate arterial stiffness, it should be noted that it does not assess the ascending aorta and part of the aortic arch. Brachial–ankle PWV (ba-PWV) is an alternative method frequently used in Asia [36] and assesses the transit of a pulse wave from the brachial artery to the ankle artery. The distance between the two measuring sites is calculated using the linear regression of body weight. Its use is limited by the different structures of the arteries found along the distance between the two arteries, which leads to ambiguity regarding its interpretation. Magnetic resonance imaging (MRI) provides accurate anatomical imaging and transit time and allows for the full-length assessment of the aorta [37]. However, costs, timing, and local availability do not allow for its use in the general population. The cardio-ankle vascular index (CAVI) is derived from the arterial stiffness index β. Its use is limited by clinical conditions, such as aortic stenosis, atrial fibrillation, and peripheral arterial disease [38]. The CAVI also allows for the assessment of both elastic and muscular arteries and, therefore, analyzes a heterogeneous phenotype. Pulse wave velocity can also be assessed indirectly through an analysis of wave reflection at the carotid and radial arteries and is quantified with the augmentation index (Aix). The Aix has different determinants: amplitude of the reflection wave, the distance to the reflected site, and the cardiac cycle. However, it has intrinsic limits, its quantification needs additional parameters (pulse pressure and systolic blood pressure), and it is often assessed together with the measurement of PWV.

Arterial stiffness has been assessed in PCOS women in different studies, as reported in Table 2. Kilic et al. assessed the CAVI in a group of 80 young women with a previous diagnosis of PCOS. Among these, 75% had hyperandrogenism, 74% had ovulatory dysfunction, and 90% had cysts on an ultrasound scan. The authors reported that PCOS women had higher CAVI values compared to controls with a key role of hyperandrogenism, which was independently associated with increased arterial stiffness [39]. Another interesting study compared PWV in PCOS women and controls at two different sites: the brachial and aorta arteries [40]. The authors found an increased arterial stiffness in the brachial artery but not at the aorta level. The possible association between PCOS and arterial stiffness could have different explanations. Indeed, one of the strongest risk factors for the stiffening of the arterial system is hypertension, which is frequently found in women with PCOS. BMI could be another possible link, considering its high prevalence in PCOS women. However, one report by Kim et al. demonstrated a negative and linear correlation between the CAVI and BMI in women with PCOS, suggesting that adiposity itself is associated with the decreased arterial stiffness [41].

Arterial stiffness has also been studied in post-menopausal women with a PCOS phenotype. Armeni et al. performed a cross-sectional study of 286 postmenopausal women and reported a higher cf-PWV in PCOS women compared to controls (9.46 vs. 8.6 m/s, *p* = 0.001) [42]. Multiple regression analysis allowed for the identification of cf-PWV as an independent predictor of PWV together with hyperandrogenism.

The possible detrimental effect of PCOS on the stiffening of the arterial system has also been evaluated in pregnant women in the study by Hu et al. [43] who assessed the carotid artery stiffness index during the three trimesters of pregnancy in 22 PCOS women. Interestingly, the stiffness index significantly increased between the second and the third trimesters. The stiffness index in the controls did not vary across trimesters and was steadily lower compared to the PCOS women (from 1.6 to 2.1-fold lower).

The pathophysiology of increased arterial stiffness in PCOS women still needs to be elucidated, but hyperandrogenism and insulin resistance may have key roles. Indeed, Agarwal et al. demonstrated the positive effect of metformin on arterial stiffness in women with PCOS [44]. In that study (randomized, double-blind, crossover design), the authors assigned 30 women to a consecutive 12 week treatment period of metformin or placebo, aiming to assess the arterial AIx and aortic PWV before and after the treatment. Metformin improved the AIx and aortic PWV but did not affect androgen levels.

Overall, the results of the study suggested an increased arterial stiffness in PCOS women, which can be reported early in young women, may further worsen during pregnancy, and can persist after menopause. Probably, the effect of pregnancy on the stiffening could be temporary due to a failure in vascular adaptation and strongly related to the pregnancy-induced hypertension [43]. However, the current data did not allow for us to draw a clear conclusion on the role of PCOS on the stiffening of the arterial system, in particular during pregnancy and after menopause, due to the lack of specific studies.

**Table 2:** Studies that evaluated arterial stiffness in women with polycystic ovary syndrome (PCOS).

Study	n		Mean Age (yrs)	Method to Assess Arterial Stiffness	Results
	PCOS	Controls			
Geronikolou [45]	19	18	13–23	PWV	Higher arterial stiffness in PCOS
Kilic [39]	80	80	22.9	CAVI	Higher arterial stiffness in PCOS, significantly related to hyperandrogenism
Kim [41]	26	59	33.3	PWV	No difference
Dargham [46]	87	663	29.3	PWV	Higher arterial stiffness in PCOS
Patel [47]	36	17	14.8	β stiffness index	Higher arterial stiffness in PCOS
Gencer [48]	52	44	24.0	β stiffness index	Higher arterial stiffness in PCOS
Rees [49]	84	95	29.8	Aortic PWV	No difference
				Brachial PWV	No difference
				Augmentation index	No difference
Armeni [42]	43	243	55.6	PWV	Higher arterial stiffness in PCOS
				Augmentation index	No difference
Zueff [50]	45	45	31.6	β stiffness index	No difference
Dessapt-Baradez [51]	14	12	26.4	Augmentation index	Higher arterial stiffness in PCOS
Moran [52]	25	27	30.0	PWV	No difference
Sasaki [53]	54	24	31.5	baPWV	Higher arterial stiffness in PCOS
Ketel [54]	22 ^#^	17 ^#^	28.6	Augmentation index and PWV	No difference
	18 ^##^	13 ^##^	30.3	Augmentation index and PWV	No difference
Cussons [55]	19	19	30.4	Augmentation index and PWV	No difference
Soares [56]	40	50	24.5	β stiffness index	Higher arterial stiffness in PCOS independently from cardiovascular risk factors
Meyer [57]	100	20	32.7	PWV	Higher arterial stiffness in PCOS
Kelly [40]	19	12	26.0	PWV brachial	Higher arterial stiffness in PCOS
				PWV aortic	No difference

Abbreviations: PCOS, polycystic ovary syndrome; N/A, not applicable; yrs, years; CAVI, cardio-ankle vascular index; baPWV, brachial to ankle pulse wave velocity. ^#^ Lean. ^##^ Obese.

### 2.3. PCOS and Atherosclerosis

Endothelium is a target organ for different metabolic risk factors. Intima-media thickness (IMT) is a widely used surrogate marker for atherosclerosis, which can be easily and noninvasively assessed with ultrasound, in particular at the common carotid level (cIMT).

Several studies reported the effect of PCOS on cIMT, but the results are not clear (Table 3). Jabbour et al. reported the results of their study, which included 41 PCOS women aged 18–34 years and 43 healthy controls. The authors assessed cIMT in both groups and found that PCOS women had an increased cIMT compared to healthy controls (0.49 vs. 0.37 mm, *p* < 0.001). Multiple regression analyses also revealed that the presence of a PCOS diagnosis was independently and positively associated with cIMT [58].

The importance of PCOS and not its isolated clinical manifestation has been highlighted in The Coronary Artery Risk Development in Young Adults Women’s Study [59]. The authors studied a cohort of women (mean age 45 years) and divided the sample in three groups: PCOS women (oligomenorrhea and hyperandrogenism), women with isolated oligomenorrhea, and women with isolated clinical or biochemical hyperandrogenism. The women with PCOS had an increased internal cIMT compared to the controls. Interestingly, both women with isolated hyperandrogenism and those with isolated oligomenorrhea had cIMT values comparable to unexposed women. Further, the authors did not find any differences at the common cIMT. It should be noted that other studies found a lack of association [60,61]. A recent meta-analysis included 96 studies (5550 PCOS women) and revealed that PCOS contributes to subclinical atherosclerosis [62], thus confirming a previous study that noted the high heterogeneity among studies [63].

Other studies also reported the effect of specific molecules that can explain the possible association between PCOS and an accelerated atherosclerosis. Adipsin, a cytokine secreted by the adipose tissue, can activate a complement pathway called factor D, which is the limiting enzyme of the alterative complement system. Furthermore, it has been found increased in some metabolic diseases and associated with endothelial dysfunction [64]. Calan et al. tested this molecule in a sample of 144 women with a diagnosis of PCOS and reported a significantly higher level of this molecule in this group compared to healthy controls, independently of androgen levels and blood pressure values [65]. Lipocalin-2 is another molecule reportedly involved in many pathological processes, including atherosclerotic plaque erosion and thrombus formation [66]. Its level has been evaluated in PCOS women in the study by Gencer et al. In that study, the authors reported an increased cIMT value in PCOS women, which was associated with lipocalin-2 in the univariate analysis [48]. However, multiple regression analysis revealed that only PCOS and not lipocalin-2 was independently associated with cIMT. Copeptin is the C-terminal portion of the precursor arginine-vasopressin, and it has been reported as a marker of cardiovascular disease [67]. One cross-sectional study compared cIMT and copeptin level in 40 PCOS women and 43 healthy controls [68]. The authors reported an increased level of copeptin in the PCOS women and a positive and linear correlation between this molecule and cIMT and free testosterone.

Overall, a correlation between PCOS and an accelerated atherosclerosis seems possible, but the data are not robust due to the small sample of the studies, which are typically case controls conducted in a clinical setting. In addition, most of the studies reported data from young-middle-aged women in which the atherosclerotic process might be somewhat still not evident. Further, the different modality of cIMT assessment might be a bias for its interpretation, as shown in Table 3 where the absolute cIMT value is double in some studies compared to others in women of a comparable age.

**Table 3:** Studies that evaluated carotid intima-media thickness in women with polycystic ovary syndrome (PCOS).

Study	PCOS	Controls	cIMT (mm)		Mean Age (yrs)	Results
			PCOS	Controls		
Atasayan [69]	65	39	0.54	0.51	23.6	No difference
Jabbour [58]	41	43	0.49	0.37	24.0	Increased cIMT in PCOS independently of main cardiovascular risk factors and hyperandrogenism
Rashad [70]	180	120	1.18	0.69	32.0	Increased cIMT in PCOS and association with intercellular adhesion molecule-1 (ICAM-1)
Bicer [71]	82	82	1.01	0.60	30.3	Increased cIMT in PCOS and association with insulin-like peptide 5
Ramoglu [72]	52	45	0.49	0.50	18–35	No difference
Gursoy Calan [65]	144	144	0.82	0.57	27.0	Increased cIMT in PCOS and association with adispin
Yilmaz [73]	25 ^#^	31 ^#^	0.23 ^#¶^	0.19 ^#¶^	21.8 ^#¶^	Increased IMT ^¶^ in PCOS
	21 ^##^	15 ^##^	0.23 ^##¶^	0.20 ^##¶^	24.5 ^##¶^	
	45 ^###^	26 ^###^	0.24 ^###¶^	0.21 ^###¶^	24.1 ^###¶^	
Taskin [74]	30	30	0.31	0.29	25.3	No difference
	30		0.30		22.9	
Macut [75]	100	50	0.54	0.47	26.3	No difference
Kahal [76]	19	17	0.51	0.48	33.9	Increased cIMT in PCOS
Aldrighi [77]	26	11	0.47	0.48	29.0	No difference
Abali [78]	37	41	0.52	0.45	25.8	Increased cIMT in PCOS
Tan [79]	83	39	0.46	0.42	27.0	Increased cIMT in PCOS
Rees [49]	84	95	0.50	0.51	29.8	No difference
Karbek [68]	40	43	0.51	0.42	23.9	Increased cIMT in PCOS
Guleria [80]	50	50	0.55	0.40	24.3	Increased cIMT in PCOS
Gencer [48]	50	44	0.61	0.50	24.0	Increased cIMT in PCOS and association with lipocalin-2
Yildir [81]	18	20	0.46	0.48	24.01	Increased cIMT in overweight PCOS
	16		0.55			

Abbreviations: cIMT, carotid intima-media thickness; PCOS, polycystic ovary syndrome; N/A, not applicable; yrs., years. ^#^ Low BMI. ^##^ Normal BMI. ^###^ High BMI. ^¶^ IMT assessed at radial artery.

### 2.4. PCOS and Cardiovascular Event

Different studies evaluated the presence of CVD outcome in women with PCOS, most of which have two main limitations. Indeed, a cardiovascular event is uncommon in young women, and therefore, studies need a wide sample size to achieve the statistical power. On the other hand, a retrospective diagnosis of PCOS in menopausal women can be underestimated. A summary of the most important studies is reported in Table 4.

In 2021, Berni et al. analyzed a wide sample of PCOS women (>174,000), aiming to establish their relative risk of myocardial infarction, stroke, angina, revascularization, and cardiovascular mortality [82]. After a 3.8 year median follow-up, the authors reported 804 incident vascular events (composite vascular event HR 1.26, 95% CI 1.13–1.41). Among these, 221 were PCOS women who developed myocardial infarction (HR 1.38, 95% CI 1.11–1.72), 319 cases of angina (HR 1.6, 95% CI 1.32–1.94), and 102 cases of revascularization (HR 1.46, 95% CI 1.06–2.02). First incident stroke and cardiovascular mortality were comparable between the two groups.

On the other hand, Lo et al. compared a cohort of 11,035 women with a previous diagnosis of PCOS to 55,175 controls without PCOS [83]. The authors found that major cardiovascular risk factors (diabetes, hypertension, dyslipidemia) were all more prevalent in the PCOS women (*p* < 0.001). However, the authors also noticed that the prevalence of coronary artery disease, cerebrovascular disease, and peripheral vascular disease was similar in both groups.

The association between PCOS and CVD is intricate, and previous studies also reported a protective effect. Indeed, Mahboobifard analyzed a cohort of 356 PCOS women and compared them to 1235 non-PCOS controls. The authors followed the participants for a median of 15.4 years and found that silent coronary artery disease was similar between the two groups [84]. However, the authors noticed that PCOS reduced the cardiovascular disease events (coronary heart disease, stroke, and cerebrovascular events) by 42% (HR 0.58, 95% CI 0.35–0.98).

A recent meta-analysis reported on the risk of cardiovascular disease and stroke events in women with PCOS [85]. The analysis included 10 cohort studies with 166,682 individuals and an average follow-up duration of 5–22 years. Compared to the controls, the PCOS women had an increased risk of any cardiovascular event (OR 1.66, 95% CI 1.32–2.08), myocardial infarction (OR 2.57, 95% CI 1.37–4.82), ischemic heart disease (OR 2.77, 95% CI 2.12–3.61), and stroke (OR 1.96, 95% CI 1.56–2.47), while no difference was observed for overall mortality and CVD-related death.

Taken together, these studies suggest an increased prevalence of CVD events in PCOS women, but these results might be limited by the presence of bias and limitations (potential inaccuracy of PCOS diagnosis, inclusion of only hospitalized PCOS women, diagnosis limited to ICD-9 codes, etc.).

**Table 4:** Cardiovascular outcome in women with polycystic ovary syndrome (PCOS).

Study	Type of Study	PCOS (n)	Control (n)	Mean Age	Mean Follow-Up	Results
Ollila [86]	Prospective	346	N/A	31 *	22 years	Increased risk of major cardiovascular event and myocardial infarction.
Berni [82]	Retrospective	174,660	174,600	29 *	N/A	Increased risk of composite cardiovascular event, myocardial infarction, angina, and revascularization.
Mahboobifard [84]	Prospective	356	1335	29.7	15.4 years	PCOS did not increase silent coronary heart disease; PCOS reduced incidence of cardiovascular disease.
Lo [83]	Retrospective	11,035	55,175	30.7	N/A	No difference in coronary artery disease, cerebrovascular disease, and peripheral vascular disease.

Abbreviations: PCOS, polycystic ovary syndrome; N/A, not applicable. * Baseline.

### 2.5. Treatment of PCOS and Reduction in CVD

PCOS does not have a specific treatment because the pathophysiology and the potential therapeutic targets are not clear. Therefore, PCOS treatment largely depends on symptomatic management and lifestyle interventions. Among these, weight reduction in obese PCOS women is a cornerstone for the achievement of better cardiovascular and metabolic outcomes.

Pharmacological treatment is mainly based on anti-diabetic agents, aiming to reduce insulin resistance, which is one of main drivers of the disease.

Among the anti-diabetic drugs, metformin is the most widely used in women with PCOS mainly due to its positive action on insulin resistance. Previous studies reported a beneficial effect on body weight reduction and also improved glucose tolerance [87,88] that, however, were lost within the first year of drug withdrawal [89].

More recently, glucagon-like peptide-1 (GLP-1) receptor agonists have been proposed as pharmacological agents for PCOS women. Beyond their weight-loss effect, GLP-1 receptor agonists have been tested for the reduction in CVD. Kahal et al. reported the results of an interventional study that enrolled 19 obese PCOS women and 17 controls [76]. Both groups were treated with increasing doses of liraglutide (0.6 mg/die for 1 week, 1.2 mg/die for one week, and then 1.8 mg/die for 6 months). The authors reported that treatment with liraglutide was associated with significant reductions in weight, insulin resistance, systolic and diastolic blood pressure, total cholesterol, triglycerides, and serum markers of endothelial function, regardless of the presence of PCOS. cIMT variation was not affected by liraglutide treatment. In 2017, the LIPT study showed that liraglutide treatment in PCOS women was associated with a significant reduction in peak thrombin concentration and lag time and a trend of reduction in plasminogen activator inhibitor-1 [90]. Subsequent studies by the same group also reported a reduction in pro-adrenomedullin and natriuretic peptide [91] and a reduction in liver fat content [92].

Gliflozins are effective drugs for the treatment of type-2 diabetes, which increase glucose excretion by inhibiting the sodium glucose cotransporter 2 in the proximal renal tubule. It is now well acknowledged that this class of drug has useful effects on congestive heart failure and renal failure.

Javed et al. tested the effect of empagliflozin on metabolic and anthropometric parameters in PCOS women who were randomized to either empagliflozin or metformin [93]. The authors reported a significant improvement of body mass index, weight, waist circumference, basal metabolic rate, and fat mass in women treated with emplagliflozin 25 mg/die in comparison to those treated with metformin 1500 mg, although no changes were observed in hormonal or metabolic parameters. The overall data on prospective effects on CVD are rather scanty. Metformin, GLP-1 receptor agonist, and gliflozin showed an improvement of anthropometric parameters and body composition, thus possibly modifying the cardiovascular risk factors. However, most of the studies have a short follow-up period, which is inadequate to estimate a clinical reduction in CVD and to draw firm conclusions.

## 3. Conclusions

Women with PCOS have an increased risk of developing major CVD risk factors during the fertile period: obesity, impaired glucose tolerance, diabetes mellitus, dyslipidemia, and metabolic syndrome. However, it is not clear whether these risks persist after the menopause period. Overall, previous studies on this topic have several biases, which limit the interpretation of their results. Indeed, it should be noted that PCOS women have different phenotypes, which can differently affect CVD. In addition, most of the studies described and analyzed a cohort of young fertile women in which the frequency of cardiovascular diseases is low. Further, longitudinal studies have a short follow-up period, which is insufficient to draw firm conclusions. Finally, pharmacological treatment is limited by the lack of specific drugs available to specifically treat PCOS. Future studies should elucidate the risk of CVD in women with PCOS, specifically in the postmenopausal years, so that women can be counseled accurately.

## Data Availability

Not applicable.
